# Radiotherapy in Patients with Cardiac Implantable Devices: A Single-Centre Retrospective Observational Analysis of Local Guidelines

**DOI:** 10.3390/jcm15082869

**Published:** 2026-04-10

**Authors:** Ellen Saghie, Roshni Manoj, Lloyd Tudor, Stuart Sandey, Catriona Buchan, Muzahir Tayebjee

**Affiliations:** Leeds Teaching Hospital Trust, Leeds LS1 3EX, UK; roshni.manoj@nhs.net (R.M.); lloyd.tudor@nhs.net (L.T.); stuart.sandey@nhs.net (S.S.); catriona.buchan1@nhs.net (C.B.)

**Keywords:** radiotherapy, pacemakers, implantable cardioverter defibrillators

## Abstract

**Background:** The aim of this study is to determine the safety of a locally implemented Standard of Practice (SOP) in patients with cardiac implantable electronic devices (CIEDs). With increasing use of radiotherapy in cancer treatment and the widespread adaptation of CIEDs, the British Heart Rhythm Society introduced new guidance in 2025. There remains ambiguity between various international, as well as manufacturer, guidelines on the management of these patients. **Methods:** This was a retrospective single-centre observational study analysing patients with CIEDs receiving radiotherapy after the implementation of our Standard of Practice in 2021. Patients were identified using the Cardiobase system. Patients were divided into non-pacemaker-dependent, pacemaker-dependent and implantable-cardioverter–defibrillator (ICD) groups. Lead sensing and impedance values were gathered pre- and post-treatment and analysed using a paired Student’s *T*-test. **Results:** A total of 320 patients were included in this study. There were no statistically significant changes in lead sensing capabilities in any of the groups pre- and post-radiotherapy with a *p* value of >0.05. There were no statistically significant changes in lead impedance in the ICD and non-pacemaker-dependent groups. Although statistically significant (*p* = 0.039), there was no clinically significant reduction in atrial lead impedance in the pacemaker-dependent cohort. **Conclusions:** From the obtained results, we can conclude that our locally implemented SOP is a safe alternative to BHRS guidelines.

## 1. Introduction

Current advances in cancer therapy accompanied by the widespread adoption of cardiac implantable electronic devices (CIEDs) has led to a clinical challenge; namely, how to deliver radiotherapy (RT) without compromising the function of CIEDs. Treatment recommendations involve a multidisciplinary team (MDT) approach involving: cardiologists, oncologists, cardiac physiologists, radiotherapists and medical physics.

Radiation, including direct ionising radiation, scatter radiation and electromagnetic interference, has the potential to interfere with CIED functioning [1,2,3]. These potential risks include inappropriate pacing or shocks, inhibition of pacing, device resets, loss of telemetry, and rarely device failure. While the risk of an adverse event is rare, they can pose serious consequences for CIED patients, particularly those who are pacing-dependent or those with implantable cardioverter–defibrillators (ICDs) [4]. The risks of CIED malfunction should also be weighed against other clinical risks, such as infection, device explantation or relocation of the device to the contralateral side.

The safety of radiotherapy in patients with CIEDs has been evaluated in several clinical studies and systematic reviews. A population-based cohort study in Denmark involving 453 patients with CIEDs undergoing radiotherapy demonstrated a low complication rate of 4.6%, with malfunctions being more likely in ICD patients. This study also demonstrated that the use of high-energy radiation beams (>10 mV) had a stronger association with device complications than the direct dose to the device. The malfunctions noted were temporary and non-life-threatening, with transient device resets being the most frequently observed complication [5]. In 2023, a meta-analysis including 3121 patients with CIEDs undergoing radiotherapy reported a complication rate of 6.6%. Similarly to the Danish study, it found more complex devices such as ICDs and cardiac resynchronization therapy defibrillators showed a higher rate of malfunction (8.2% and 19.8% respectively) when compared to standard PPMs (4.1%). The risk factors identified in this meta-analysis included device type, exposure to high-energy photon beams and proximity to radiation field. Notably, the cumulative radiation dose to the device demonstrated an inconsistent correlation with the risk of malfunction [6]. A separate meta-analysis was carried out in 2023 and involved 2454 patients. This analysis once again found ICDs were more vulnerable to malfunction and the use of high-energy beams (>10 mV) significantly increased this risk. Interestingly, this study showed no significant difference between chest radiotherapy and other treatment sites, suggesting scatter radiation and neutron effects could play a role in device malfunction [1].

The British Heart Rhythm Society (BHRS) released a guidance document in January 2025, replacing its previous 2015 guidelines, for the management of patients with CIEDs receiving radiotherapy [7]. These guidelines first highlight the requirement for early MDT involvement and mandates reporting CIED dose in the radiotherapy planning phase, with a recommended maximum dose to the device of 5 Gy. Patients are then to be risk-stratified into three distinct groups: low-risk (patients with pacemakers that are not pacing-dependent), medium-risk (patients with pacemakers that are pacing-dependent or patients with ICDs that are not pacing-dependent) and high-risk (patients with ICDs that are pacing-dependent, or patients with a dose to CIED of >5 Gy). These guidelines advise a staff member appropriately trained in arrhythmia recognition with immediate life support (ILS) training be present to monitor the patients during their first treatment. For the low-risk category, a CIED check is recommended at the start and end of treatment. For the medium-risk group, device reprogramming to a non-sensing mode or magnet placement is recommended for pacing-dependent patients and a magnet to disable anti-tachycardia therapies is recommended in ICD patients; continuous cardiac monitoring is recommended throughout. A CIED check at the start, middle, and end of treatment is also advised. For the final high-risk group, the BHRS advises continuous cardiac monitoring as well as reprogramming devices pre and post each fraction of radiotherapy, with magnet placement during treatment. Weekly checks are advised after the device receives a dose of >5 Gy.

Despite these guidelines, recommendations from CIED manufacturers remain inconsistent in terms of the maximum dose the device can be exposed to and contribute to clinical uncertainties [8,9]. A recent in vitro study irradiating modern CIEDs under clinically relevant high-dose-rate beam regimes found no significant change in pacing output when devices were placed in asynchronous mode [10].

Separate from this are the financial pressures and chronic staff shortages, both in the National Health Service (NHS) and worldwide, that make implementation of the guidelines into clinical practice a challenge. A recent Dutch study examined the barriers and facilitators to clinical guideline implementation and identified some key barriers. These barriers included: limited resources (e.g., limited time and workforce capacity and insufficient funding), lack of infrastructure, the complexity and volume of guidance, and limited local engagement [11]. In the NHS alone over 100,000 vacancies across clinical roles were reported in 2025 with funding growth remaining low, contributing to ongoing strains on the service [12,13].

To try and reduce demand on clinical staff, The Leeds Teaching Hospital Trust (LTHT) developed its own Standard of Practice (SOP) in 2021, which is still in place today. There are significant differences in the SOP when compared to BHRS guidelines. The objective of this paper is to conduct a retrospective observational study, assessing safety outcomes, on patients with CIEDs who received radiotherapy after the implementation of this SOP in 2021.

## 2. Materials and Methods

**Figure 1:**
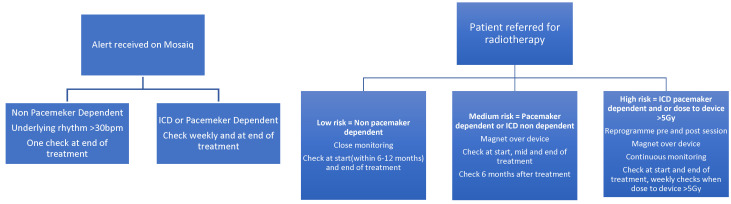
Flow charts comparing recommendations between locally implanted SOP (**left**) and BHRS guidelines (**right**).

Following collaboration between the cardiology and radiotherapy department, the SOP was first introduced and implemented at LTHT in 2021, prior to the recent BHRS 2025 guidelines. Based on our SOP, patients receiving radiotherapy are identified via MOSAIQ, our local oncology database, and an alert is sent by the oncology team to our cardiac physiologists. Patients with an underlying rhythm were classified as low-risk and their devices are checked only at the end of treatment. Patients who had no underlying rhythm or those who had ICDs implanted had weekly device checks performed. All patients received an end-of-treatment device check. Our SOP does not mandate device reprogramming or magnet placement during radiotherapy in pacing-dependent or ICD patients. A full copy of the SOP is available in the Supplementary Material. Figure 1 below demonstrates a brief comparison of our SOP to the current BHRS guidelines.

We carried out a single-centre retrospective observational study in November 2025 to analyse the safety of our SOP. We used the Cardiobase system (Version 8.3.373.0), which is an electronic database used at LTHT to manage patient data in the cardiology department. Patients receiving radiotherapy were identified via the Cardiobase system by filtering for ‘radio’ or ‘radiotherapy’ in the visit-type search bar. Patients identified were then input into PPM+ (PPM+ 2025), which is the online electronic medical record system used at LTHT. Initial data collected using PPM+ included: site of radiotherapy, dose delivered to body and as well as any recorded complications. All patients who received radiotherapy prior to 2021 were excluded from this study. Patients who did not have their post-radiotherapy device check at our institution were also excluded. The authors then looked further into patients who were characterised as medium- and high-risk based on BHRS risk stratification. The lead sensing and lead impedance values pre- and post-therapy were analysed in these patients via Cardiobase. We also analysed the lead impedance and sensing data that were available to us from the low-risk non-pacemaker-dependent patients. Lead sensing and impedance values were compared pre- and post-radiotherapy using a paired Student’s T-test in all 3 groups. Patient demographics were not analysed as they were not variables likely to materially influence the study outcomes.

Patients that were identified via Cardiobase were then input into Mosaiq (Version 3.22), our local oncology database. Any documented dose delivered to the device was extrapolated from this database and included in the dataset.

This study was reviewed by our local Research and Innovation Department and classified as service evaluation.

## 3. Results

A total of 320 patients were identified that fulfilled the inclusion criteria. The cohort was divided into three groups based on device type and pacing dependency. A total of 188 (188/320) patients had pacemakers in situ and were not pacing-dependent; 57 (57/320) patients were pacemaker-dependent; 75 (75/320) patients had ICDs implanted, with 2 of the 75 being pacemaker-dependent.

**Figure 2:**
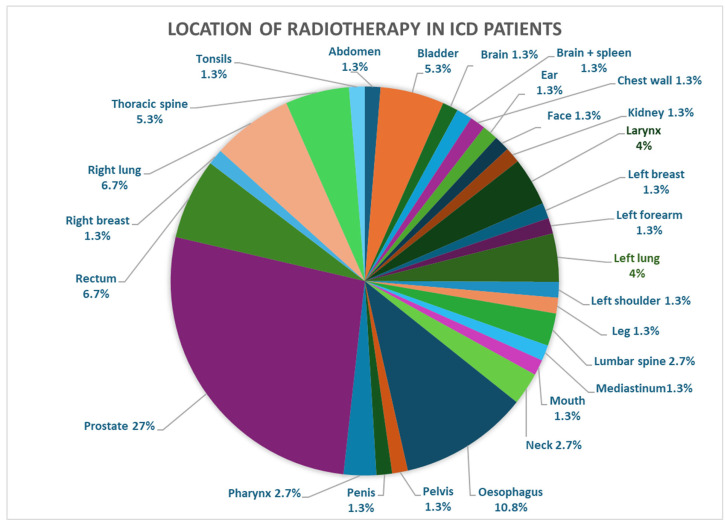
Location of radiotherapy in patients with implanted ICDs.

**Figure 3:**
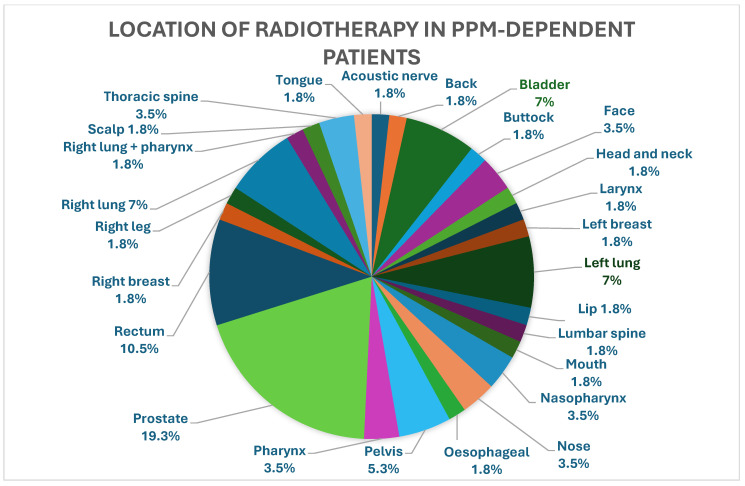
Location of radiotherapy in PPM-dependent patients.

**Figure 4:**
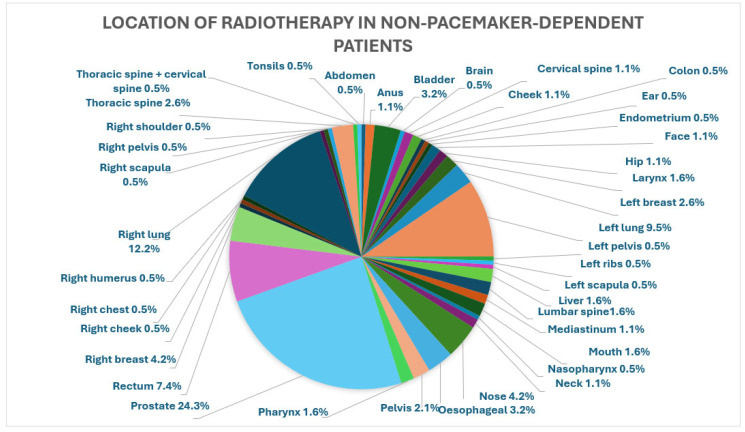
Location of radiotherapy in non-pacemaker-dependent patient with implanted PPMs.

The majority of radiation fields were in regions distant from the CIED: 26 (26/320) patients received radiotherapy to their left breast or left lung, 2 (2/320) patients had devices on the right side, and one received therapy to the left breast and the other to the left forearm. Figure 2, Figure 3 and Figure 4 below depict a breakdown of the various locations of the radiotherapy received in the three groups.

The device dose was documented in patients whose CIED was within 10 cm of the treatment field. In total, 73 (73/320) patients had a documented device dose. This included 24 (24/189) patients who were non-pacemaker-dependent, 30 (30/75) patients with ICDs and 19 (19/57) pacemaker-dependent patients. The highest documented dose to the device was 7.7 Gy. Three patients (3/320) had a documented dose of >5 Gy. No adverse events were noted in these patients. The mean device dose was 1.16 Gy.

A single clinical complication was recorded among the study population. One patient in the non-pacemaker-dependent group was found to have a spike in their right ventricle (RV) lead impedance requiring placement of a new RV lead. This patients’ device was not in the radiotherapy field and they had no documented device dose.

The analysis of the pacing-dependent group is demonstrated in Table 1. There were no statistically significant changes pre- and post-radiotherapy in RV impedance (*p* = 0.108), left ventricle (LV) impedance (*p* = 0.098), P-wave sensing (*p* = 0.204), or R-wave sensing (*p* = 0.236). A statistically significant decrease was noted in atrial impedance (mean pre-radiotherapy = 466.8, mean post-radiotherapy = 445.4; *p* = 0.039); however, this mean difference was small and not clinically significant. No intervention was required in this cohort of patients due to changes in atrial impedance.

The ICD group demonstrated no statistically significant changes across any pacing or high-voltage parameters, as shown in Table 2. The atrial (*p* = 0.994), RV (*p* = 0.625), and LV (*p* = 0.115) lead impedances remained stable. High-voltage resistance (HVR) impedance (*p* = 0.109) and high-voltage superior vena cava (HV-SVC) impedance (*p* = 0.366) showed no statistically significant variation. The P wave (*p* = 0.843) and R wave (*p* = 0.397) amplitudes also remained stable pre- and post-radiotherapy.

Consistent with the higher risk groups, the low-risk non-pacing-dependent group showed similar lead parameters, as demonstrated in Table 3. The atrial (*p* = 0.584), RV (*p* = 0.513), and LV (*p* = 0.868) impedances showed no statistically significant variation pre- and post-treatment. The P wave (*p* = 0.350) and R wave (*p* = 0.432) amplitudes also showed no significant degradation.

Overall, there was no evidence of clinically significant changes in device parameters associated with the current SOP at LTHT.

## 4. Discussion

The purpose of this study was to assess the safety of the locally implemented SOP at LTHT. This study showed no statistically significant difference in device parameters pre- and post-radiotherapy treatment. From the patients followed up at our centre, there were no adverse events noted from time of radiotherapy to time of data collection apart from the one patient who required a new RV lead. Given that this patient’s device was not in the radiation field, it is unlikely there is a causative link between lead failure and radiotherapy treatment.

The guidelines for managing patients with CIEDs receiving RT have evolved over time; however, there remains ambiguity in practice. Guidelines published by the European Society of Cardiology (ESC) in 2022 recommend deactivating the sensor in rate-adaptive PPMs [10]. The ESC also states that deactivating anti-tachycardia therapies in ICD patients is not an essential recommendation and is infrequently performed in practice despite consensus statements suggesting this [14,15,16]. The guidelines also vary regarding monitoring of CIEDs post-RT. Some countries recommend checks at 1, 3 and 6 months, with others recommending them at 1 and 6 months [14,17,18,19].

With advancements in medicine and an ageing population, the implantation of CIEDs has been on the rise. With this comes the importance of reducing device complications. The need for device re-intervention and potential complications including device infection place a burden on the healthcare system and have an impact on patient morbidity and mortality.

Based on current practice, the SOP is a safe and an easily implementable alternative to the BHRS guidelines. Based on the SOP, patients are risk-stratified into two categories: the first is standard non-dependent pacemaker patients and the second is patients with ICDs or those who are pacemaker-dependent. We defined standard patients as patients who have an underlying rhythm of above 30 beats per minute (bpm) with no symptoms and recommend only an end-of-treatment check in these patients. For patients who are pacing-dependent and those with ICDs, we recommend one check following the first fraction of radiotherapy and weekly checks thereafter, with an end-of-treatment check. Our SOP does not recommend any programming changes to devices in terms of switching to non-sensing modes, and device therapies were not turned off either by reprogramming or with magnet placement. Unlike the BHRS guidelines, the SOP does not mandate a member of staff trained in arrhythmia recognition and immediate life support be present at radiotherapy sessions. This in turn places less stress on hospital resources.

As this was a retrospective study, we relied on existing clinical documentation which may not have been collected with research objectives. This can potentially lead to missing or incomplete data, which in turn limits us from drawing definitive conclusions. The study evaluates outcomes under an SOP without a control group subjected to the BHRS guidelines. Therefore, direct comparative conclusions about its superiority or inferiority cannot be made. While the overall cohort contained 320 patients, the subgroups for specific lead parameters are smaller. This could limit the statistical power to detect small, potentially clinical, changes. Dose-to-device values were only available in patients who had a CIED within 10 cm of the therapy field. It would be helpful to know this information in all patients as there is a suggestion that scatter radiation can also lead to device malfunction [1]. The study focused on immediate pre- and post-treatment device checks. The effects of radiotherapy were not assessed in the long term due to a large cohort of patients being followed up in other centres post completion of radiotherapy.

## 5. Conclusions

The results demonstrated that our locally implemented SOP is a safe alternative to BHRS guidelines and places less strain on services. Analysis of long-term adverse events would be beneficial to further strengthen this. The authors do, however, acknowledge that this is a retrospective study with small subgroup sizes.

## Figures and Tables

**Table 1:** Comparison of device parameters pre- and post-radiotherapy in pacing-dependent patients.

	N	Mean	Standard Deviation	T Value	*p* Value
Atrial impedance pre-RT (ohms)	35	466.8	91.5	−2.15	0.039
Atrial impedance post-RT (ohms)	35	445.4	85.6		
RV impedance pre-RT (ohms)	39	571.8	138.8	−1.65	0.108
RV impedance post-RT (ohms)	39	558.9	113.7		
LV impedance pre-RT (ohms)	4	596.0	179.9	−2.37	0.098
LV impedance post-RT (ohms)	4	533.3	179.6		
P wave pre-RT (mV)	33	2.824	1.775	−1.30	0.204
P wave post-RT (mV)	33	2.676	2.011		
R wave pre-RT (mV)	38	4.11	6.12	1.20	0.236
R wave post-RT (mV)	38	5.26	7.28		

RV = Right ventricle, LV = left ventricle, RT = radiotherapy.

**Table 2:** Comparison of device parameters pre- and post-radiotherapy in ICD patients.

	N	Mean	Standard Deviation	T Value	*p* Value
Atrial impedance pre-RT (ohms)	55	434.7	95.3	−0.01	0.994
Atrial impedance post-RT (ohms)	55	434.6	82.1		
RV impedance pre-RT (ohms)	71	461.2	130.3	−0.49	0.625
RV impedance post-RT (ohms)	71	458.7	137.6		
LV impedance pre-RT (ohms)	34	713.2	228.4	−1.62	0.115
LV impedance post-RT (ohms)	34	695.8	224.0		
HVR impedance pre-RT (ohms)	66	63.08	15.98	−1.62	0.109
HVR impedance post-RT (ohms)	66	60.89	15.90		
HV SVC impedance pre-RT (ohms)	15	62.87	11.44	−0.93	0.366
HV SVC impedance post-RT (ohms)	15	60.13	10.88		
P wave pre-RT (mV)	54	2.783	1.588	−0.20	0.843
P wave post-RT (mV)	54	2.754	1.531		
R wave pre-RT (mV)	71	12.61	12.81	−0.85	0.397
R wave post-RT (mV)	71	11.35	5.74		

RV = Right ventricle, LV = left ventricle, RT = radiotherapy, HV = high voltage, SVC = superior vena cava, HVR = high-voltage resistance.

**Table 3:** Comparison of device parameters pre- and post-radiotherapy in non-pacemaker-dependent patients.

	N	Mean	Standard Deviation	T Value	*p* Value
Atrial impedance pre-RT (ohms)	55	512.1	129.9	−0.55	0.584
Atrial impedance post-RT (ohms)	55	507.2	120.9		
RV impedance pre-RT (ohms)	67	588.7	199.7	0.66	0.513
RV impedance post-RT (ohms)	67	610.4	344.0		
LV impedance pre-RT (ohms)	8	714.4	153.6	−0.17	0.868
LV impedance post-RT (ohms)	8	709.9	131.5		
P wave pre-RT (mV)	50	3.079	1.655	−0.94	0.350
P wave post-RT (mV)	50	2.946	1.650		
R wave pre-RT (mV)	62	11.113	5.513	−0.79	0.432
R wave post-RT (mV)	62	10.797	5.424		

RV = Right ventricle, LV = left ventricle, RT = radiotherapy.

## Data Availability

No new publicly available data due to patient confidentiality. The raw data supporting the conclusions of this article will be made available by the authors on request.

## Supplementary Materials

The following supporting information can be downloaded at https://www.mdpi.com/article/10.3390/jcm15082869/s1.

## Abbreviations

The following abbreviations are used in this manuscript:

CIED	Cardiac Implantable Electronic Devices
ICD	Implantable Cardioverter–Defibrillator
PPM	Pacemaker
BHRS	British Heart Rhythm Society
SOP	Standard of Practice
MDT	Multidisciplinary team
RT	Radiotherapy
ILS	Immediate Life Support
NHS	National Health System
LTHT	Leeds Teaching Hospital Trust
RV	Right Ventricle
LV	Left Ventricle
SVC	Superior Vena Cava
HV	High Voltage
HVR	High-Voltage Resistance
ESC	European Society of Cardiology
BPM	Beats Per Minute
