# Supplementary material for: Radiotherapy in Patients with Cardiac Implantable Devices: A Single-Centre Retrospective Observational Analysis of Local Guidelines
Source: J Clin Med. 2026 Apr 10;15(8):2869. doi: 10.3390/jcm15082869 (PMC13115797; doi:10.3390/jcm15082869)
Supplement: Supplementary file 1 [file jcm-15-02869-s001.zip › jcm-4195873-supplementary.pdf]

The Leeds Teaching Hospitals NHS Trust<br>**Clinical Physiology**<br>**Standard Operating Procedure**	Title<br>Scope<br>Version<br>Date<br>SOP ID	STANDARD OPERATING PROCEDURE<br>Management of Cardiac Devices During Radiotherapy

Details:

Author: Philip Smith, Specialist Cardiac Physiologist and Zara Patterson, Highly Specialised Cardiac Physiologist

SOP Pages: 9

Version No: 1.0

Effective Date of SOP: 04/01/2021

Approval:

**Version No. SOP being approved**	**Name of person approving SOP**	**Date**	**Signature of person approving SOP**
1.0	Michael Lupton	03/02/21	
1.0	Andrew Hogarth	03/02/21	

Distribution & Storage:

Distribution to:

Clinical Cardiac Physiology Workforce, Leeds Teaching Hospital NHS Trust
Consultant Cardiologists, Leeds Teaching Hospital NHS Trust
Radiotherapy Workforce, Leeds Teaching Hospital NHS Trust
Consultant Oncologists, Leeds Teaching Hospital NHS Trust

Location of Document:

Paper: Head of Clinical Physiology's Office, Leeds General Infirmary

Electronic: I : Cardiol on 'lgijub'\1.Clinical Physiology\SOPs

CONTENTS

Front page		Page	01
Contents		Page	02
Section A	Introduction	Page	02
Section B	Applicability	Page	03
Section C	SOP: Management of Cardiac Devices During Radiotherapy	Page	03
1.	Staffing requirements	Page	03
2.	Cardiac / Respiratory Arrest Alarm	Page	03
3.	Resuscitation Equipment	Page	03
4.	Patient Inclusion	Page	03
5.	Information flow for requests	Page	03
6.	Information required from Radiotherapy to Cardiac Physiologist	Page	03
7.	Information required by the Cardiac Physiologist	Page	04
8.	Risk stratification	Page	04
9.	Patient appointments	Page	04
10.	Complications	Page	05
11.	Recovery and / or unwell patients	Page	05
12.	Obtaining urgent clinical advice	Page	05
13.	Discharge of patient	Page	05
14.	Reporting	Page	05
15.	Archiving	Page	05
16.	Health & Safety	Page	05
17.	Audit	Page	06
18.	Training	Page	06
19.	Approval of a new or amended SOP	Page	06
20.	Review of SOPs	Page	06
Section D	Definitions / Abbreviations Used in Reports	Page	06
Section E	References and Contacts	Page	07
Section F	Appendices and Protective Marking	Page	08

Section A Introduction

Historically, implantable cardiac devices have been considered at significant risk of electromagnetic interference (EMI) being detected as cardiac signals, potentially leading to adverse events such as withholding of pacing therapy or inappropriate treatment. It was previously thought that EMI may be detected in device patients during Radiotherapy.
Research over the past ten years has shown that, with modern devices, EMI is no longer a clinically relevant issue in the vast majority of cases. In addition, there have been revisions to the clinically safe maximum dose a device should be exposed to and beam energy delivered is now also taken into account when managing patients with cardiac devices. This SOP has been produced with the aim of

streamlining radiotherapy and cardiology procedures in the management of these cases, defining responsibilities and ensuring the service is modernised and in line with evidence based practice.

Ideally communication between departments should take place using email, to provide an audit trail of communication. Telephone calls should be documented contemporaneously and letters should be sent using the Epro system. All parts of the procedure should be paper-free where possible.

Section B Applicability

This SOP is relevant to all staff involved in the direct care of oncology patients undergoing radiotherapy treatment who have a pacemaker or ICD insitu. This includes doctors requesting the treatment, the clinical staff delivering the treatment, the administrative staff in cardiology and oncology as well as the cardiac physiologists.

Section C SOP: Management of Cardiac Devices during Radiotherapy

1. Staffing requirements

Staff required: Specialist Cardiac Physiologist (band 6) or above, with training and experience to understand the risks to a devices patient inherent in radiotherapy and how they should be mitigated. Medical Physicists experienced in calculating doses from RT and who understand the risks posed to / potential complications caused by cardiac devices. Administrative and booking staff in radiotherapy and cardiology. Oncologists requesting radiotherapy. Registered staff should have appropriate experience and CPD to demonstrate their skills. Staff should have access to Mosaiq, PPM and Epro.

2. Cardiac / Respiratory Arrest Alarm

If a devices patient has an arrest in the Radiotherapy Department, the Cardiac Physiologist at SJUH should be alerted after the crash team has been called (via bleep if necessary but ideally by telephone call to the Cardiorespiratory Department at SJUH to ensure the message is delivered promptly). No other changes to standard procedures. Staff present should be aware that the patient has a cardiac device.

3. Resuscitation Equipment

Standard adult crash trolley should be available in all areas. This should be equipped and checked as per Trust standards. Cardiac Physiologist should attend with the appropriate programmer and equipment to interrogate the device if required.

4. Patient Inclusion

This SOP applies to any patient with an implanted pacemaker or defibrillator who is undergoing radiotherapy at LTHT.

5. Information flow for requests

See “Flowchart for Processing Radiotherapy Enquiries at SJUH”.

6. Information required from Radiotherapy to Cardiac Physiologist

- Patient identifiable information (i.e. Patient name, date of birth, NHS number)
- Device follow up centre
- Medical Physics will alert if beam energy in excess of 10MV or device exposure >5Gy

It is expected that any change to treatment outside of the standard (such as high dose rates to the device) will be discussed. This is covered on the electronic shared pre-procedure checklist in appendix 9.

7. Information required by the Cardiac Physiologist

The following must be obtained before the patient can be risk stratified and radiotherapy can proceed.

- Device make and model
- Most recent follow-up details and test results
- Device dependency status and underlying rhythm
- Other relevant information such as therapies delivered by an ICD or any problems with the system
- Device location.

External queries should follow Trust information governance guidance and ideally should be made by email for traceability. Received information from external sources should be entered into Cardiobase to ensure it is available to other Physiologists.

8. Risk stratification

The Specialist Cardiac Physiologist will be required to risk stratify the patient and to communicate the management strategy to Radiotherapy Booking via the electronic booking form on Mosaiq. Guidance is found in the attached document labelled “Guidance for Risk Stratification of Radiotherapy Patients with Cardiac Devices”. Located on the I drive.

Patients with pacemakers who are not dependent will be low risk and will require “standard management” (as defined in Section 9).

Those with ICDs and/or who are pacemaker dependent fall into a higher risk category and subject to “weekly management” (as defined in Section 9).

Beam energies above 10MV or total device doses >5Gy are rare but the risk of device malfunction increases at this stage. Medical Physics will alert the Cardiac Physiologist to any such patients and this will discussed with a Consultant Cardiologist to determine an appropriate management strategy.

An appropriate letter should be sent to the patient detailing when they need to see the Cardiac Physiologist. A template letter (appendix 2) is attached as a guide. An information sheet should be sent with this letter (appendix 6).

Decisions and the rationale behind the management strategy must be documented on Cardiobase under the appropriate Pacemaker or ICD module in a manner that allows another Cardiac Physiologist to follow the instructions and plan without clarification.

The outcome of the risk stratification should be added to the shared form on Mosaiq.

9. Patient appointments

Patient’s radiotherapy treatment appointments **must** be made during the working day of the on-site, cardiac devices Physiologist (Monday - Friday 0830-1630). Patients with a PPM or ICD should **not** be treated without a suitably qualified cardiac physiologist on site with the appropriate equipment. Remote monitoring will be used, if available.

Patients on standard management must be seen by Cardiac Physiology **at the end of treatment.**

Weekly patients must be seen by the Physiologist **after the first treatment** and **weekly**, as well as **at the end of treatment**. The first treatment check is to look for any signs of EMI using VHR detection, SIC counters, etc. The patient should be asked if they had any symptoms during the procedure. Questions or uncertainties should be solicited and addressed. A full set of standard measurements is not required as up to date information should be available before starting radiotherapy. Appendix 5 provides full technical guidance.

Interim appointments (weekly) should involve a full device check as per the appropriate SOP for the patient's device, as should end of treatment checks.

Any reprogramming that the Physiologist feels is necessary can be performed during these appointments. The patient's usual follow up centre should be informed of any permanent changes to programming.

Decisions on the patient's schedule should be documented on the shared form on Mosaiq and on Cardiobase.

10. Potential complications

In the unlikely event that the radiotherapy treatment has had an adverse effect to device function, the following may be noted; programming changes which were not made using a programmer (due to memory corruption), increased thresholds, significant changes in the battery status or any significant loss of device function. Power on resets should be noted but only if they repeat frequently should they be considered an issue. Any reset which results in a loss of programmed parameters or a change in function (such as backup pacing) should be considered a complication and discussed with Medical Physics. In most cases, closer monitoring will be sufficient action. Evidence of permanent damage to the pulse generator should result in consulting the manufacturer and cardiology consultant for consideration of replacement. Complications are discussed in detail in Appendix 5 – technical guidance.

11. Recovery and / or unwell patients

Radiotherapy patients are frequently unwell or exhausted. If the Cardiac Physiologist has concerns then they should contact radiotherapy. Porters should be offered where they may help.

12. Obtaining urgent clinical advice

If there are concerns then escalation to the appropriate specialty is required. This may be Radiotherapy, the Consultant Oncologist / Cardiologist, a more senior Cardiac Physiologist or the appropriate administrative team. Contacts can be found through AVA or on the attached contact sheet (appendix 1)

13. Discharge of patient

The patient will be discharged back to routine follow up at the cessation of radiotherapy. Documentation will be sent to the patient's follow up centre.

14. Reporting

Where possible this should be electronic. Paper files may be used to store programmer print outs where this is not supported electronically. Cardiobase should be used to document all patient contacts, actions taken, results, outcomes, etc. Epro should be used to send any letters. Emails should be used where possible. The shared form on Mosaiq should be completed in full prior to any radiotherapy being performed. When signed off as complete, this will be automatically sent to PPM during the next overnight synchronisation.

15. Archiving

Electronic systems specified already have archive functions. Paper records for the final check will need placing in the appropriate pacing file or sending, with the Cardiobase records, to the appropriate centre. All changes to Mosaiq documents are recorded. The shared form is sent to PPM when complete.

16 .Health & Safety

All LTHT Health and Safety guidelines must be adhered to regarding length of time looking at a computer screen and ergonomics of chair and desk height to ensure a comfortable working environment. Programmers should be handled in accordance with manual handling guidance. Patients should not be transferred unless absolutely necessary and can remain in their wheelchair if they arrive in one.

17. Audit

This procedure will be audited on a regular basis to maintain quality assurance and to ensure problems with the process are addressed. Adverse events will result in a complete audit of the patient in question.

18. Training

As a minimum, the SOP and adjoining documentation must be read by relevant staff and this should be documented on the hard copy. It is not expected that all staff will have to read every associated document, only those relating to their area of practice. The staff undertaking this procedure should have the appropriate end point exam completed and is aware of escalation procedures if a concern about a patient arises.

19. Approval of a new or amended SOP

Approval or suspension of a new or amended SOP can only be performed by:

- Head of Clinical Physiology
- Clinical Director / Clinical Lead

Confirmation of the process is performed by signing and dating of the front cover of the SOP.

Production of a new SOP may be requested by any qualified member of staff. The request will be reviewed / approved by the Head of Clinical Physiology who will confirm the requirement of the new SOP.

20. Review of SOPs

Head of Clinical Physiology will be responsible for ensuring all SOPs are reviewed regularly (and at least every 2 years) in order that they are in line with current processes, guidelines and regulations.

The introduction of a new SOP will carry version 1.0 once it has been approved for the first time.

When the SOP has been reviewed and needs to be amended, this will carry the version number 2.0 and increase chronologically after each further amendment / approval.

Associated documents (appendices) are marked as fixed or fluid documents. Fixed documents must not be changed without formal review of the SOP as a whole and review of the latest evidence. This is because changing these documents will have potential medico-legal consequences and even isolated changes may impact the systematic protections which have been drawn from a particular evidence base, leading to clinical consequences. These must be read only or require a password to edit.

Fluid documents are those which require constant updates to remain useful and where not keeping these updated is more likely to cause medico-legal issues. Rationale for each document being labelled fluid is given in the appendix list.

Section D Definitions / Abbreviations used in reports

A/E	Accident and Emergency
AF	Atrial fibrillation
AFl	Atrial Flutter
APB	Atrial Premature Beats
BPM	Beats per minute
CP	Cardiac Physiologist
ECG	Electrocardiogram
EP	Electrophysiology
GP	General Practitioner
Gy	Grays
HSCP	Highly Specialised Cardiac Physiologist
ICD	Implantable Cardioverter-Defibrillator
ILS	Immediate Life Support
LGI	Leeds General Infirmary
LINAC	Linear Accelerator
LTHT	Leeds Teaching Hospitals Trust
MRI	Magnetic Resonance Imaging
MV	Megavolts
PAF	Paroxysmal atrial fibrillation
PAC	Premature atrial contraction
POR	Power on Reset
PPM	Permanent Pacemaker
RT	Radiotherapy
SJUH	St James's University Hospital
SOP	Standard Operating Procedures
SPR	Specialised Registrar
SVT	Supra Ventricular tachycardia
VE	Ventricular Ectopic
VT	Ventricular Tachycardia
VF	Ventricular Fibrillation

Section E References and Contacts

References:

1. HRS Expert Consensus Statement on Magnetic Resonance Imaging and Radiation Exposure in Patients with Cardiovascular Implantable Electronic Devices. HRS Online (May 2017)
2. Risk of device malfunction in cancer patients with implantable cardiac device undergoing radiotherapy: a population-based cohort study. PubMed Link (March 2015)
3. Management of radiation oncology patients with a pacemaker or ICD: a new comprehensive practical guideline in The Netherlands. Dutch Society of Radiotherapy and Oncology (NVRO). PubMed Link (Nov 2012)
4. Radiotherapy-induced pacemaker and implantable cardioverter defibrillator malfunction. PubMed Link (May 2009)

5. Radiotherapy in patients with pacemakers and implantable cardioverter defibrillators: a literature review. EP Europace Link (April 2016)

6. Policy for the management of radiotherapy patients with cardiac implantable electronic devices (Northern Centre for Cancer Care / Cardiology Joint Policy, Newcastle Upon Tyne Hospitals. 2018).

7. Work Instruction For Management Of Patients With A Cardiac Implantable Electronic Device (Liverpool Heart and Chest Hospital. 2019).

8. Management of radiotherapy patients with implanted cardiac pacemakers and defibrillators: A Report of the AAPM TG-203, December 2019

Contacts:

See attached contact sheet (appendix 1)

Section F Appendices and Protective Marking

1. Contact Sheet **FLUID**
2. Example Patient Letters **FLUID**
3. Guide for Risk Stratification and Management for Physiologists **FIXED**
4. Administrative Workflow for Radiotherapy Patients with Cardiac Devices **FLUID**
5. Cardiac Physiologist Technical Procedure for Radiotherapy Patients **FIXED**
6. Patient Information Sheet **FIXED**
7. Responsibilities **FLUID**
8. Advice for Radiotherapists Treating Patients with Cardiac Devices **FIXED**
9. Guide for Completing Pre-Treatment Checklist on Mosaiq **FLUID**
10. Associated documents protective markings and rationale **FIXED**

Documents marked as fluid may be changed without re-approval of the entire SOP as they need regular updates to remain useful and changes will not have an impact on the clinical decisions or treatment of the patient.

Documents marked as fixed must NOT be changed without formal revision of the SOP as a whole and review of the evidence.

Flowchart for processing Radiotherapy pacemaker enquiries at SJUH

Alert received on Mosaiq

↓

If this is a patient external to LTHT, the follow up location should be provided. They should be contacted and information requested.[1] If an LTHT patient, the patient's Cardiobase file should be reviewed.

↓

Standard PPM

NAD on recent checks

ULR >30bpm without Sx

Standard management

One check at end of treatment

ICD or Dependent PPM

No ULR @ VVI 30 for >30seconds and/or any ICD

Weekly management

Check after first fraction

Check Weekly and EOT

Other concerns

Mostly covered in SOP technical guidance

Create appropriate management plan

↓

Complete the cardiology section on the shared form on Mosaiq detailing the management plan. Document on Cardiobase.

↓

Letter to patient on Epro, explaining the device has been assessed and the plan for its management (standard letters part of SOP, remember to customise if required). Also send standard information sheet with directions, etc.

↓

When RT appointments are available for the patient, enter in diary (i.e. date of final check for standard management or date of first fraction for weekly management).

↓

Checks as per management plan / SOP

Any issues should be managed as per the SOP and escalated if required.

↓

Full device check after final treatment regardless of management strategy. Send the report to the follow up centre, ideally by PDF into email.

↓

END
